# Few-Layer Graphene-Based Nanofluids with Enhanced Thermal Conductivity

**DOI:** 10.3390/nano10071258

**Published:** 2020-06-28

**Authors:** Samah Hamze, Nawal Berrada, David Cabaleiro, Alexandre Desforges, Jaafar Ghanbaja, Jérôme Gleize, Dominique Bégin, Florentin Michaux, Thierry Maré, Brigitte Vigolo, Patrice Estellé

**Affiliations:** 1 Laboratoire de Génie Civil et Génie Mécanique, Université de Rennes, F-35000 Rennes, France; samah.hamze@univ-rennes1.fr (S.H.); dacabaleiro@uvigo.es (D.C.); thierry.mare@univ-rennes1.fr (T.M.); 2 Institut Jean Lamour UMR7198, CNRS, Université de Lorraine, F-54000 Nancy, France; nawal.berrada@univ-lorraine.fr (N.B.); alexandre.desforges@univ-lorraine.fr (A.D.); jaafar.ghanbaja@univ-lorraine.fr (J.G.); brigitte.vigolo@univ-lorraine.fr (B.V.); 3 Dpto. Física Aplicada, Facultade de Ciencias, Universidade de Vigo, 36310 Vigo, Spain; 4 Laboratoire de Chimie et Physique Approche Multi-échelles des Milieux Complexes, Université de Lorraine, F-57000 Metz, France; jerome.gleize@univ-lorraine.fr; 5 Institut de Chimie et Procédés pour l’Energie, l’Environnement et la Santé (ICPEES) CNRS-University of Strasbourg, 25, rue Becquerel, 67087 Strasbourg, CEDEX, France; dominique.begin@unistra.fr; 6 Laboratoire d’Ingénierie des Biomolécules, Université de Lorraine, 2, avenue de la Forêt de Haye, 54500 Vandoeuvre-lès-Nancy, France; florentin.michaux@univ-lorraine.fr

**Keywords:** few-layer graphene, propylene-glycol/water, nanofluids, thermal conductivity, temperature effect, concentration influence, theoretical prediction

## Abstract

High-quality graphene is an especially promising carbon nanomaterial for developing nanofluids for enhancing heat transfer in fluid circulation systems. We report a complete study on few layer graphene (FLG) based nanofluids, including FLG synthesis, FLG-based nanofluid preparation, and their thermal conductivity. The FLG sample is synthesized by an original mechanical exfoliation method. The morphological and structural characterization are investigated by both scanning and transmission electron microscopy and Raman spectroscopy. The chosen two-step method involves the use of thee nonionic surfactants (Triton X-100, Pluronic^®^ P123, and Gum Arabic), a commercial mixture of water and propylene glycol and a mass content in FLG from 0.05 to 0.5%. The thermal conductivity measurements of the three FLG-based nanofluid series are carried out in the temperature range 283.15–323.15 K by the transient hot-wire method. From a modeling analysis of the nanofluid thermal conductivity behavior, it is finally shown that synergetic effects of FLG nanosheet size and thermal resistance at the FLG interface both have significant impact on the evidenced thermal conductivity enhancement.

## 1. Introduction

The growth in energy consumption pushes us to make an energy transition to low carbon generation and design more efficient utilization approaches, which requires a research effort focused on the development of innovative, intelligent, durable, and effective solutions [1]. Heat transfer plays an important role in many industrial processes, such as electronic and thermoelectric devices, refrigerators, heat exchangers, solar energy systems, heating and cooling of buildings, among others [2,3,4,5]. In such applications, the low inherent thermal conductivity of most conventional thermal media can become the main limitation in improving performances and reducing energy consumption [6]. Because solids have intrinsic thermal properties higher than conventional heat transfer fluids, like water, ethylene glycol, and engine oil; it was proposed to disperse millimetric and micrometric solid particles within standard fluids to enhance their thermal properties. The idea of “nanofluids” appeared in 1995 by Choi et al. [7], who introduced the use of nanosized particles, which makes the solutions more stable than when bigger particles are used because of the size effect and the Brownian motion of the nanoparticles in the fluid [8]. Nanoparticles do not only stay suspended much longer in the base fluid than micron-sized suspensions [9]; as compared with microparticles, their surface to volume ratio of nanoparticles is much higher (~1000 times) [6]. This, in turn, allows for obtaining much better thermal properties in the case of nanofluids rather than colloidal suspensions of microparticles or the base fluids alone. Over the last decades, many scholars focused on the investigation of nanometric particle suspensions [10,11,12,13,14,15,16,17,18]. The key issues in nanofluid research are to prepare stable solutions and increase the thermal conductivity of the liquid, without a significant increase in viscosity that could penalize pumping power [19].

Many factors have been reported to affect the thermal conductivity of a nanofluid, such as temperature [20,21], particle size [22,23], particle shape [24], concentration of nanoparticles [25], and addition and type of surfactant [26,27]. For that, the researchers studied different types of nanofluids based on various thermal fluids and containing a wide selection of nanoparticles, such as metal oxides [28,29,30,31,32], metallic nanoparticles [33,34], or carbon nanomaterials [35,36,37,38,39,40,41]. Since 2004, when Novoselov et al. [42] first isolated graphene, this material has been proposed for many interesting applications, owing to its excellent physical, chemical, and mechanical features [10]. The researchers investigated many nanofluids based on graphene, as this carbon allotrope exhibits high intrinsic thermal properties, with thermal conductivities of several order higher than other types of nanomaterials [43]. For example, Gupta et al. [44] compared different water based nanofluids that differed by the nanoparticle type, and showed that their graphene based nanofluids had the highest thermal conductivity enhancement, with a maximum value equal to 27% at 0.2 vol.%. Gao et al. [45] obtained an increase of the thermal conductivity of their graphene nanofluids based on ethylene glycol, ethylene glycol:water (1:1), and water, by 4.6, 18, and 6.8%, respectively (in all three cases with a 0.15 wt.% content of graphene). On the other hand, functionalized graphene nanosheets were dispersed by Vallejo et al. [46] in a mixture of propylene glycol:water (30:70) wt.%. The thermal conductivity results showed an enhancement about 16% at the maximum weight concentration 1%. In addition, Seong et al. [35] prepared 0.1 wt.% of graphene water-based nanofluids (7 nm of thickness and size about 40 nm) where sodium dodecyl sulfate (SDS) and sodium dodecyl benzene sulphonate (SDBS) were added separately as surfactants. The investigated graphene:surfactant ratios were 1:3, 1:2, 1:1, 2:1, 2:1, and 3:1. Their measurements have shown that the thermal conductivity decreased with increasing the amount of surfactant in the sample. In addition, the authors found that, with 1:3 and 1:2 of graphene:surfactant, nanofluids with SDS had a lower conductivity than with SDBS, while, with the graphene:surfactant ratios lower than 1:1, SDS showed a thermal conductivity greater than for SDBS, and a maximum improvement was achieved in the case of the graphene:SDS ratio of 2:1. Bahaya et al. [38] focused on the graphene nanosheets (diameter 5 μm and thickness 3 nm) dispersed in water with the addition of gelatin to prevent the sedimentation. The authors prepared their nanofluids with nanoparticle concentrations up to 0.014% in volume. A maximum relative thermal conductivity equal to 1.43 was obtained at the higher concentration. Graphene based nanofluids can also differ in terms of treatment applied to graphene and it is generally admitted that the thermophysical properties depend on these treatments and dispersion methods. While previous studies mainly focused on graphene oxide (GO), reduced GO (rGO), or functionalized graphene, only few studies have been reported about the use of few layer graphene to produce and characterize nanofluids.

Sun et al. studied the exfoliation of pristine graphite into few layer graphene (FLG) while using acrylate polymer solutions in low boiling point alcohols [47]. The authors proved the effectiveness of acrylate polymers to exfoliate a few layers graphene and obtain stable suspensions in both ethanol and isopropanol. Thermal conductivity measurements at different temperatures from 283.15 to 333.15 K showed a quasi-constant enhancement (around 25%) for low nanofluid volume concentration of 0.055% in ethanol. Amiri et al. investigated a water based highly crumpled few layer graphene (HFLG) nanofluids where the nanoparticles were mixed with Gum Arabic (GA) by a GA:HFLG ratio of 0.5:1 [48]. With increasing the weight concentration of the nanofluid from 0 to 0.01%, an enhancement of 42.5% was obtained at 323.15 K. In addition, the authors found that the thermal conductivity increased by 8.6, 20.8, 23.8, and 21.9% for deionized water and nanofluids concentrations 0.001, 0.005, and 0.01 wt.%, respectively, with temperature increasing from 283.15 to 323.15 K. Amiri et al. focused on mono-layer graphene nanoparticles dispersed in water with different weight contents between 0.005 and 0.01% [49]. They found an increase in thermal conductivity values with increasing temperature from 283.15 to 323.15 K. The results showed an enhancement in the thermal conductivity of nanofluids by more than 25% for different temperatures and weight fraction of 0.01%. Alawi et al. recently presented a new method to prepare covalent-functionalized FLG based on a thermal treatment using pentaethylene glycol [50,51]. Water-based nanofluids containing various concentrations of functionalized FLG (0.025–0.1 wt.%) were characterized in terms of temporal stability and thermo-physical properties. A maximum enhancement in the thermal conductivity of 31% was observed at 323.15 K for the highest nanoparticle concentration (0.1 wt.%).

We report, in this study, the preparation and the comprehensive characterization of FLG prepared from an ecofriendly mechanical exfoliation method presented here for the first time as a contribution of graphene-based nanofluid development for heat transfer applications, and the use of FLG in particular that still remains weakly investigated. The as-produced high-quality FLG is used for the production of graphene based-nanofluids prepared with a commercial heat transfer fluid, namely Tyfocor^®^ LS, which is a mixture of propylene glycol:water (40:60) wt.% [52]. These nanofluids were produced when considering Triton X-100, Pluronic^®^ P-123, and Gum Arabic as surfactants. The stability at rest of the nanofluids was analyzed by Turbiscan and their thermal conductivity was measured and analyzed with regard to the kind of the used surfactant, temperature (283.15–323.15 K), and concentration of graphene (0.05, 0.1, 0.25, and 0.5 wt.%). Finally, the thermal conductivity of the FLG-based nanofluids was analyzed with relevant models while taking the influence of several parameters, such as the average length, interfacial thermal resistance, thickness, or flatness ratio of FLG, into consideration in order to provide a further insight that could help to understand the reasons behind the thermal conductivity enhancements in such prepared graphene-based nanofluids.

## 2. Materials and Methods

### 2.1. Materials

FLG was synthesized by a mechanical exfoliation method that was assisted by tannic acid. Typically, 200 mg of tannic acid (Sigma–Aldrich, Lyon, France) was dissolved in 400 mL of deionized water, and then 400 mg of expanded graphite (provided by Mersen, Courbevoie, France) was added in the beaker in which a sonication probe (Branson Ultrasonics^TM^ Sonicator 400 W) was plunged. Sonication (80 W, 50 kHz, continuous mode) was performed for 4 hours at 298.15 K.

The prepared FLG pre-dispersed in the aqueous tannic solution was washed with DW before freeze-drying. Typically, 250 mL of the FLG solution was placed in a vacuum filtration set-up and then filtered on a membrane of 0.45 µm porosity (Merck Millipore, Darmstadt, Germany) and washed five times with 250 mL of DW. After freeze-drying, the obtained FLG powder was ready for analysis and nanofluid preparation. The FLG graphene produced for each batch and following this method is around 300 mg, which means that the production yield is around 80%.

Graphite powder, namely TIMCAL TIMREX^®^ SFG6 Primary Synthetic Graphite (Timcal Inc., Westlake, OH, USA), was used for comparison with the as produced FLG for Raman spectroscopy. A commercial water-propylene glycol-based heat transfer fluid, namely Tyfocor^®^ LS (referred to Tyfocor in the following figures and text) was gently provided by Viessmann S.A. and it was used for nanofluid preparation. Tyfocor^®^ LS consists in a mixture of propylene glycol:water with 40:60 wt.% [52]. This heat transfer fluid was selected, as it is a ready-to-use industrial material containing corrosion and ageing inhibitors. Nonionic surfactants such as Triton X-100 (Sigma Aldrich, Germany), Pluronic^®^ P123 (Sigma Aldrich, Saint-Quentin Fallavier, France), and Gum Arabic (Acros Organics, Illkirch, France) have been selected for the nanofluid preparation, because they have been reported to have good ability to disperse carbon nanomaterials [53,54]. Actually, the use of surfactants is an effective approach for efficiently unbundling nanoparticles and ensuring nanofluid stability without altering the pristine graphene structure [55]. Ionic surfactants have proven effective to provide good dispersibility of carbon-based nanoparticles in water. However, those ionic surfactants may lead to the formation of foam inside thermal facilities, which, in turn, may reduce the effective surface of heat transfer and, consequently, thermal performance. Unlike some ionic surfactants, non-ionic surfactant, such as Gum Arabic, do not create foam when agitated [53]. Among the surfactants containing non-ionic block copolymers, Triton X series (Triton X-100 or Triton X-405, for instance) [56,57,58] or Gum Arabic (GA) [57,59,60,61] have been the most common when preparing surfactant-aid graphene nanofluids. Moreover, as compared with other surfactants (SDS, CTAC, or PVP), Triton X-100 surfactant aqueous solutions seem to be more effective to enhance the thermal performance of pulsating heat pipes, for example [62]. Pluronic^®^ P-123 was proven to be an effective surfactant to prepare graphene dispersions in water, methanol, ethanol, 1-hexano, or ethylene glycol (in comparison with equivalent graphene:surfactant ratios of SDS, CTAB, or Triton-X). In addition, such non-ionic surfactant is biodegradable and economical and does not lead to any foam formation, which makes it an interesting alternative to design graphene nanofluids [63].

For each surfactant, nanofluids with 0.5 wt.% of FLG were first prepared from the two-step method by adding the desired amount of surfactant to Tyfocor^®^ LS, respectively 1 wt.%, and then introducing the right amount of FLG powder within this mixture. Afterwards, the nanofluid sample with 0.5 wt.% in FLG was sonicated while using a probe sonicator (Bioblock Scientific Vibra cell 75042, 125 W with a pulse mode 2 s ON/1 s OFF) for 5 × 15 min. controlling also the temperature of the sample to avoid overheating effects. This nanofluid was then diluted with Tyfocor to obtain nanofluid samples with lower FLG concentrations: 0.25, 0.1, and 0.05 wt.%. These samples were also sonicated following the same procedure after dilution. Consequently, the ratio of surfactant/FLG was similar and equal to 2 for the three prepared nanofluid series. A similar procedure was used for each surfactant (Triton X-100, Pluronic^®^ P123 or Gum Arabic).

### 2.2. Characterization Techniques

Scanning electron microscopy (SEM) analysis was carried out using a XL30 S-FEG apparatus. Transmission electron microscopy (TEM) and high-resolution TEM (HRTEM) observations were performed using a JEM-ARM 200F apparatus at a low accelerating voltage (80 kV) to avoid possible damaging by the electron beam. Holey carbon grids (200 mesh size) were used, so that the image contrast could be improved for this all-carbon nanomaterial. Approximately 30 images were taken at different areas for each sample to guarantee statistically representative observations. Fast Fourier Transform (FFT) and Inverse FFT (IFFT) were calculated on the selected area of FLG images by using Digital micrograph software.

A LabRAM HR 800 micro-Raman spectrometer was used for Raman spectroscopy analysis. The incident wavelength was a red light at λ = 632.8 nm. For the analysis, the FLG was gently dispersed in ethanol by means of a sonication bath and then deposited on a glass slide. At least five spectra were recorded for each sample. For data analysis, a baseline was first subtracted and the height of the D band was divided by that of the G band to calculate the I_D_/I_G_ intensity ratio.

In an original way, nanofluids stability against sedimentation was followed by multiple light scattering measurements using a Turbiscan Classic MA2000 apparatus (Formulaction, Toulouse, France) using a pulsed near infrared light source (λ = 850 nm). Two synchronous detectors measured transmitted and backscattered light upon sample height by several scans by up movements (every 40 µm) all along a glass cylindrical cell until the top of the sample (5–7 cm). In the case of nanofluid dispersions, only the transmitted light intensity has been followed upon sample height, since no backscattered light has been detected due to black particles light absorption. Additionally, only the nanofluids with the two lowest FLG concentrations were analyzed. For the high FLG concentration nanofluids, the light transmission was too low to obtain reliable measurements. Transmitted intensities all along the sample height have then been recorded upon time. Scans have been recorded during five days after particles dispersion (t_0_). The first scan recorded at t_0_ has been removed to the followings in order to highlight the system evolution upon time using Turbisoft software (version 1.2.2, FormulAction, Toulouse, France). Subsequently, the relative percentage of transmitted intensity (ΔBS) upon sample height has been reported and its evolution upon time has also been visualized while using this software. A sedimentation phenomenon is then characterized by an increase of the transmitted light at the top of the sample upon time until every particle settles. The transmitted signal then remains constant. The sedimentation rate was calculated by considering that the height of the sediment at five days (longest time) corresponded to the final sedimentation state. The Turbiscan measurements have been performed at room temperature.

The density of FLG was measured at ambient temperature while using a Quantachrome gas pycnometer Ultrapyc 1200e (Quantachrome Instruments, Anton Paar, Boynton Beach, FL, USA) working with helium under a pulse mode suitable for powders. Before starting measurements, the device was calibrated to an accuracy of ± 0.002 cm^3^ using two standard steel spheres (1.0725 cm^3^ each one). An absolute average deviation (AAD) of 0.66% was found between the volume measurements with the instrument and their real volume (2.145 cm^3^). For the measurements of FLG density, a micro cell of 4.5 cm^3^ was used and the FLG sample was carefully weighted with a precision balance. A true density value of 1.82 ± 0.02 g/cm^3^ was finally obtained. This value will be considered later in order to evaluate the thermal conductivity of FLG and the FLG volume fraction used in thermal conductivity models for nanofluids.

The thermal conductivity of the dry FLG nanopowder was obtained by means of a Direct Thermal Conductivity-meter DTC-25 (TA instruments, New Castle) working with the guarded heat flow meter technique according to the Standard Test Method proposed in the ASTM E1530 [64]. This device is suitable for studying thermal conductivities from 0.1 to 20 W·m^−1^·K^−1^ at an ambient temperature. A manual press B13142 Graseby Specac (Specac Ltd., Orpington, UK) was used to compact the dry nanopowder and create a disk 50 mm in diameter. Thermal conductivity results with this device have an experimental accuracy of 6% and repeatability of 2%. Additional details regarding this instrument or the followed experimental procedure can be found in [65].

**Figure 1:**
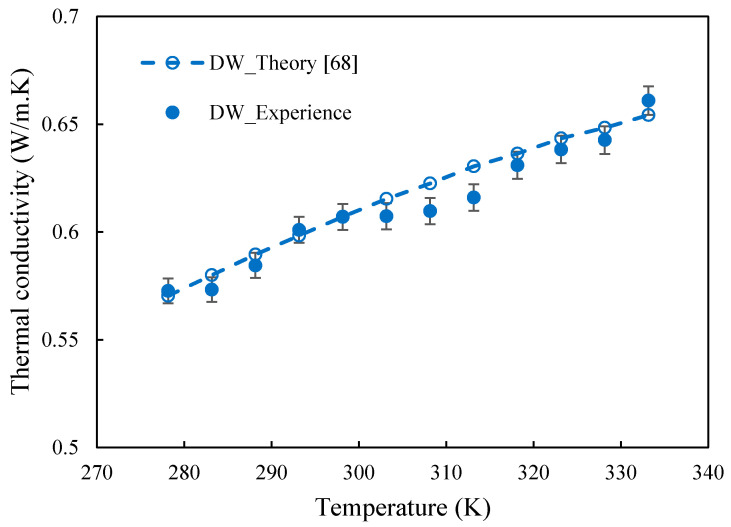
Comparison between the experimental thermal conductivity of DW and reference values extracted from [68]. Error bars indicate average absolute deviation between experiments and reference values in the temperature range 278.15–333.15 K.

The thermal conductivity of both base fluids and nanofluids was evaluated with a THW-L2 device (Thermtest Inc., Richibucto Road, NB, Canada) using the transient short hot-wire method according to the ASTM D7896 standard. This device has been designed to measure the thermal conductivity of liquids in the range 0.01-2 W·m^−1^·K^−1^ under short time of measurement to avoid convection. The full description of the experimental set-up has been previously reported in [66,67] and a similar experimental procedure has been followed. A power supply varying between 90 and 110 mW, to reach a temperature rise of 1.2 K, has been applied here to samples with a time measurement of 1.5 s for thermal conductivity evaluation. This value has been classically calculated in the linear region of the temperature enhancement versus time in logarithm scale. The temperature probe and the wire of the sensor have been calibrated with DW (DIUF, CAS 1132-18-5, Fisher Chemical, 0.599 W·m^−1^·K^−1^ at 293.15 K) before measurements [67]. Once the device calibrated, the thermal conductivity of deionized water has been measured in the temperature range 278.15-333.15 K, as shown in Figure 1, in order to evaluate the experimental uncertainty of the device. These data have been compared to reference values [56]. A really good agreement was achieved, with an average absolute deviation (AAD) around 1%, as evidenced by Figure 1. As for DW, thermal conductivity values of base fluids and nanofluids presented in the following consist in an average of at least six measurements with 5 min. between each test for each tested temperature. A total of 25 different samples, including pure Tyfocor^®^ LS, base fluids (three Tyfocor+surfactant series containing 0.1, 0.2, 0.5 and 1 wt.% concentrations of either Triton-X100, Triton X-100, Pluronic^®^ P-123, Gum Arabic), and nanofluids (three FLG+Tyfocor+surfactant series containing 0.05, 0.1, 0.25, and 0.5 wt.% loadings of FLG) have been tested. This leads to at least 750 data points.

## 3. Results and Discussion

### 3.1. Few-Layer Graphene Synthesis and Characterization

**Figure 2:**
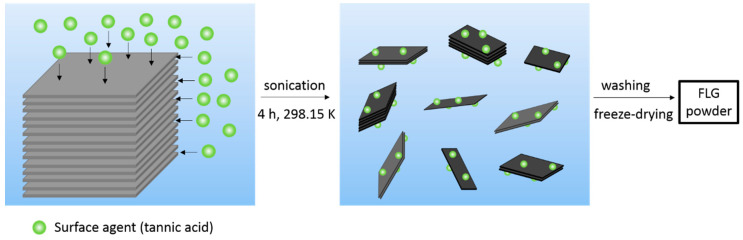
Schematic of the mechanical exfoliation method in aqueous medium assisted with tannic acid.

When compared to the Hummers’ method [69], bulk synthesis of graphenic materials from mechanical exfoliation of graphite or expanded graphite [70] assisted by sonication produced graphene with better structural quality. Aqueous media were advantageously non-toxic and easier to handle as compared to the methods using organic solvents [71]. Surfactants, such as polycyclic aromatic hydrocarbons, have shown high ability to assist graphene exfoliation due to strong π–π interactions [72,73]. For the first time in the literature, here we propose assisting aqueous exfoliation of expanded graphite by biosourced surfactants, such as tannic acid, in a whole green process. Tannic acid, a phenolic acid (C_76_H_52_O_46_), has the double positive role of i) inducing π–π interactions between its C_6_ rings and those of the graphene surface assisting that way tannic acid intercalation between graphene sheets together with ii) facilitating graphene nanosheet dispersion in water thanks to the OH groups that acid tannic belongs (see Figure 2). Such a green procedure can be very suitable to nanofluid preparation requiring a relatively high amount of nanomaterials.

**Figure 3:**
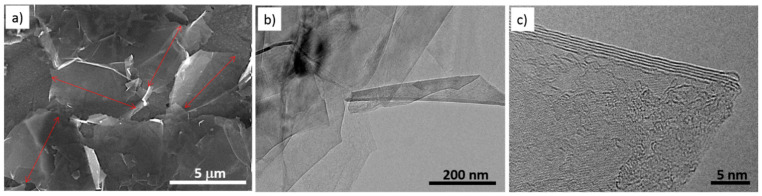
Scanning electron microscopy (SEM) (**a**), Transmission electron microscopy (TEM) (**b),** and (**c**) images of the synthesized few-layer graphene (FLG).

Figure 3 shows the SEM and TEM images of the synthesized graphenic material. SEM (Figure 3a) and TEM low magnification observation images (Figure 3b) both show that the prepared material is under the form of very thin sheets of about 3–5 layers with a thickness typically between 1 and 2 nm (typical image shown in Figure 3c), meaning that the used exfoliation method preferentially produced FLG [74]. The mean lateral size of the FLG sheets was found around 5 μm on average, as evidenced in Figure 3a.

**Figure 4:**
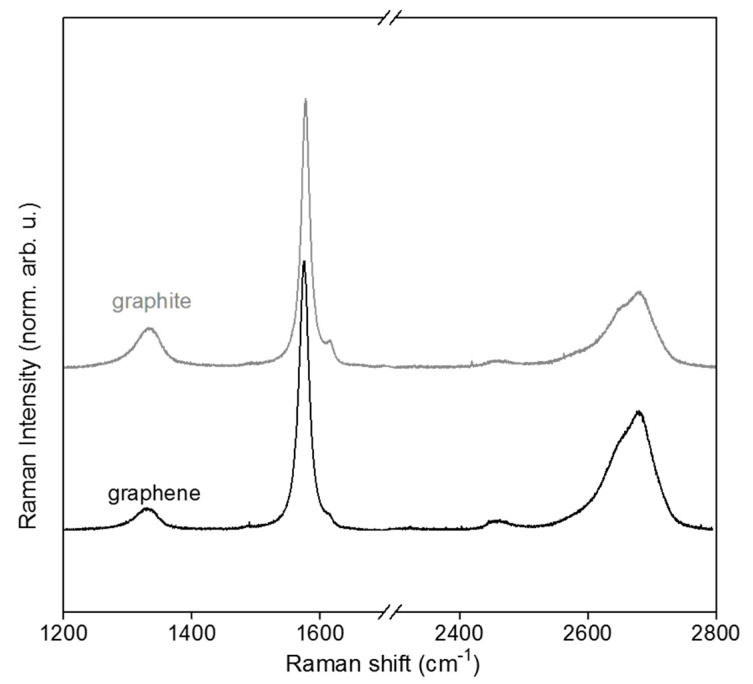
Raman spectra of FSG6 “graphite” and produced few layer “graphene” FLG.

**Figure 5:**
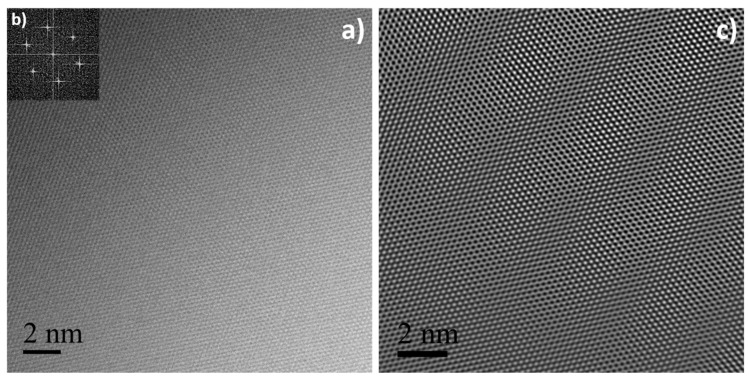
High-resolution TEM (HRTEM) image of FLG (**a**), corresponding Fast Fourier Transform (FFT) (**b**), and Inverse FFT (IFFT) (**c**).

HRTEM and Raman spectroscopy are commonly used as complementary techniques to finely probe the structural quality of nanostructured carbon materials. Figure 4 shows a typical spectra of high-quality graphite (SFG6) and the FLG used in this study. The most intense peak is the G band that originates from the sp^2^ bonded carbon atoms of the hexagonal lattice of a graphitic structure, and it is well visible around 1580 cm^−1^ [75]. The D band, around 1350 cm^−1^, is related to sp^3^ defects present in the sp^2^ carbon atom network [76]. I_D_/I_G_ is relatively low for both samples and the I_D_/I_G_ of graphite is higher than that of FLG, meaning that this latter was of high structural quality. The 2D band, around 2700 cm^−1^, of higher intensity for FLG is in agreement with the few layer nature of the used graphenic material (Figure 3c). In agreement with Raman spectroscopy, HRTEM, FFT, and IFFT images (Figure 5a–c, respectively) have shown the used FLG has an excellent structural quality with its well noticeable honeycomb carbon atom network [77].

### 3.2. Stability at Rest of the Prepared FLG-Based Nanofluids

**Figure 6:**
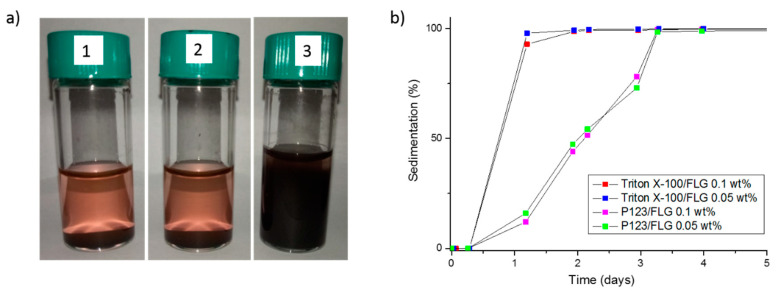
(**a**) Photos of FLG based nanofluids with 0.1 wt.% of FLG and (1) Triton X-100, (2) Pluronic^®^ P123, and (3) Gum Arabic as surfactant six days after their preparation. (**b**) Sedimentation level from Turbiscan for FLG based nanofluids with Triton X-100 and Pluronic^®^ P123 for 0.05 and 0.1 wt.% of FLG.

Static stability of the FLG-based nanofluids was followed over time by both visual observations and Turbiscan analysis. Turbiscan allowed for measuring sedimentation evolution within the FLG based nanofluids that were prepared with Triton X-100 and Pluronic^®^ P123 (Figure 6b). In the case of these two nanofluid series, the sedimentation behavior was not modified by the FLG concentration. Complete sedimentation was more rapid for Triton X-100 than for Pluronic^®^ P123. Sedimentation of the FLG nanoparticles was completed after one day with Triton X-100 while with Pluronic^®^ P123, sedimentation of FLG was reached three days after the nanofluid preparation (Figure 6b). Unfortunately, a reliable analysis was not possible with Gum Arabic, while this nanofluid was visually observed more stable than the two others. It should be finally mentioned that the thermal conductivity values of nanofluids reported thereafter are obtained after sample preparation. Their dispersion state is stable for the whole measurement run period. Indeed, if a sedimentation was occurring during the measurements, a decrease of the thermal conductivity would be detected during the experiment, as has been observed for unstable GO-based nanofluids [78].

### 3.3. Thermal Conductivity of FLG and Nanofluids

According to the parallel cylinder model of porous media [79], the “apparent” thermal conductivity of the FLG nanosheets, denoted $k_{app}$, was estimated as the ratio between the values directly measured by the thermal conductivity-meter k and the apparent volume fraction of FLG nanosheets φ in the studied disk by the following Equation (1):

(1) $$k_{app}=\frac{k}{\phi }$$

The apparent volume fraction of FLG, φ, in the compacted disk was calculated from the ratio between the apparent density of the FLG nanosheets in the disk (ratio between the FLG mass used to produce the disk and the theoretical volume of the disk) and the experimental density of the FLG powder, determined previously from gas pycnometry. An apparent thermal conductivity of ~12.0 W·m^−1^·K^−1^ was obtained with compacted volume fractions of ~0.33–0.34. These values are higher than the effective thermal conductivities of 1.37 or 10.7 W·m^−1^·K^−1^ measured by Vallejo et al. [46,80] for other graphene samples. Such a difference might be attributed to the less aggressiveness exfoliation process that was used in this study (aqueous medium assisted with tannic acid).

Thermal conductivity of the prepared FLG nanofluids with different weight concentrations (0.05–0.5%) were measured between 283.15 and 323.15 K in the presence of Triton X-100, Pluronic^®^ P123 and Gum Arabic as surfactants. The results are presented in the following by first discussing the effect of the surfactant addition alone on the thermal conductivity of Tyfocor^®^ LS. Subsequently, the effect of the surfactant, temperature, and nanoparticle content on the thermal conductivity of the FLG-based nanofluids are analyzed. Finally, thermal conductivity enhancement of FLG-based nanofluids are compared to some theoretical models in an attempt to explain the observed behavior.

**Figure 7:**
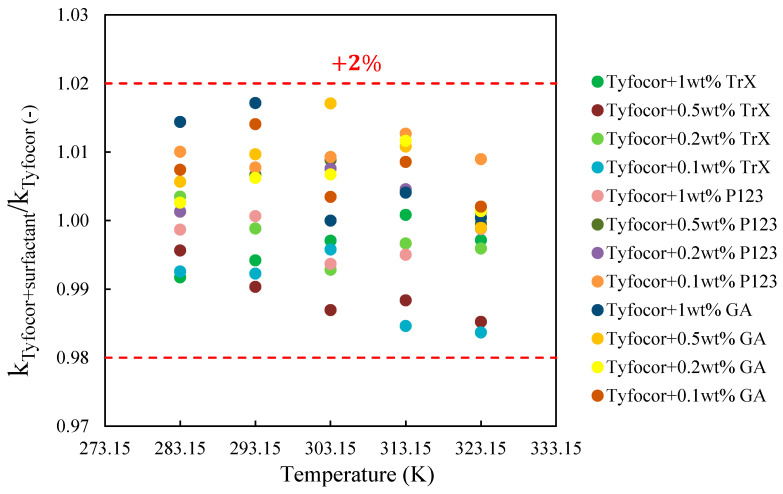
Thermal conductivity of Tyfocor^®^ LS alone and the base fluids with the three different surfactants used:Triton X-100 (TrX), Pluronic^®^ P-123 (P123), Gum Arabic (GA) for the given surfactant concentration between 283.15 and 323.15 K.

As expected, an enhancement of thermal conductivity of Tyfocor^®^ LS and base fluids was observed with the temperature rise. For the base fluids, this enhancement did not vary with the concentration of surfactant and, over the 40 K temperature domain, the thermal conductivity of the base fluids with Triton X-100, Pluronic^®^ P123, and Gum Arabic increased by 6.4, 7, and 6.3%, respectively. The thermal conductivity of Tyfocor^®^ LS alone and the ratio between the base fluids of the three series (Tyfocor^®^ LS + Triton X-100, Tyfocor^®^ LS + Pluronic^®^ P123 and Tyfocor^®^ LS + Gum Arabic) where the concentration of surfactant was varied between 0.1 and 1% in mass is shown in Figure 7. In all cases, deviations between thermal conductivities of Tyfocor^®^ LS with surfactant and Tyfocor^®^ LS only remained within the 2%. Taking the experimental uncertainty into account, the presence of surfactant (whatever the concentration used) did not significantly impact the thermal conductivity of Tyfocor^®^ LS over the tested temperature range of 283.15–323.15 K.

**Figure 8:**
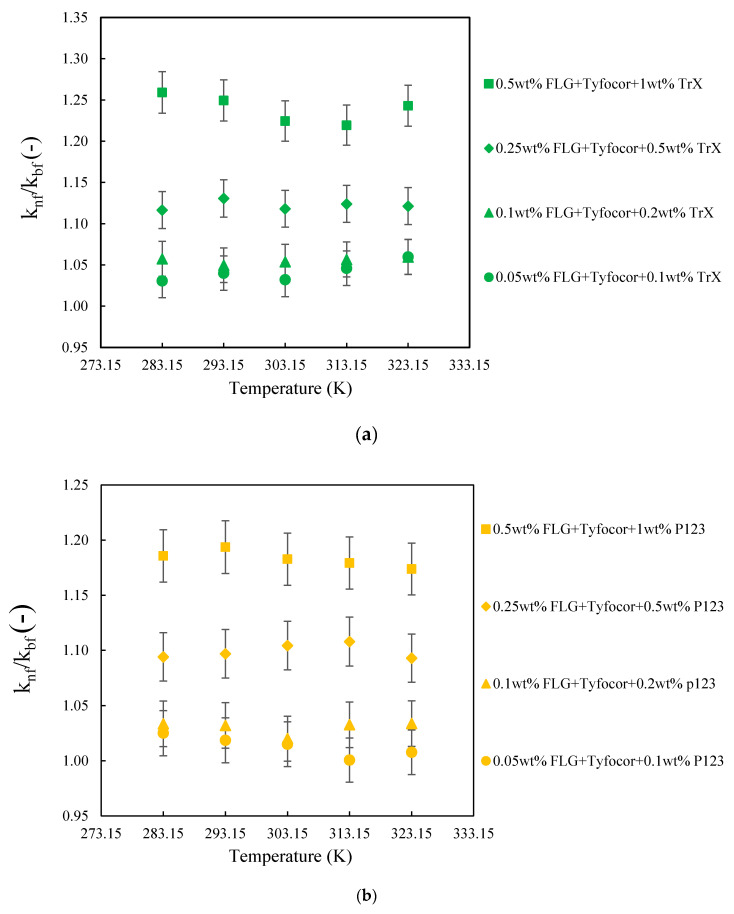

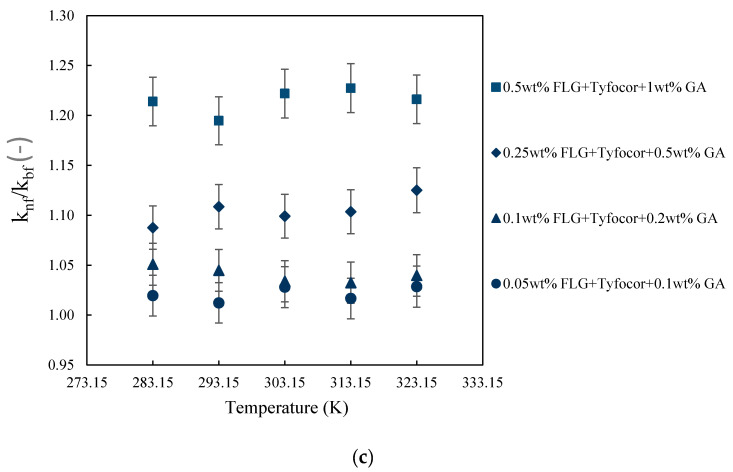
Thermal conductivity ratio of the FLG-based nanofluids with TrX (**a**), P123 (**b**), and Gum Arabic (GA) (**c**), as surfactant as function of FLG concentration and temperature between 283.15 and 323.15 K. Error bars indicate 2%.

Like for the base fluids, the thermal conductivity of the prepared nanofluids was observed here to increase with both temperature and FLG concentration in agreement with the literature. Figure 8 presents the thermal conductivity ratio for the three series of nanofluids as function of temperature and FLG content. The results show that the thermal conductivity of the base fluids and those of the nanofluids increased with temperature and the relative thermal conductivity of all nanofluids was quasi-constant with the variation of the temperature, within the experimental uncertainty. A similar trend has been previously reported in other works [81,82,83]. It is widely known that the concentration of the nanoparticles plays an important role in improving the thermal conductivity and then heat transfer of nanofluids. As already mentioned, the studied concentration of FLG nanosheets varies between 0.05 and 0.5% in mass. Figure 8 shows that the thermal conductivity of nanofluids increased with FLG content. For the FLG concentration of 0.05, 0.1, 0.25, and 0.5 wt.%, the nanofluid thermal conductivity increases by 4.2, 5.5, 12.2, and 23.9%, respectively, as compared to the corresponding base fluids when using Triton X-100 as a surfactant. The thermal conductivity enhancements are 1.3, 3.0, 9.9, and 18.3% in the case of Pluronic^®^ P-123. Finally, the observed increases in conductivity reach 2.1, 4.0, 10.5, and 21.5% with Gum Arabic. Due to the slight change of thermal conductivity enhancement with temperature, the previous values corresponded to the average of the results in the studied temperature range. A weak dependence in the type of the used surfactant with regards of thermal conductivity enhancement was also observed. One can notice that the enhancement presently reported for the higher FLG concentration is better than the values obtained by Agromayor et al. [82], who reported an enhancement of 12% at the mass concentration of 1% of graphene in water at 313.15 K, or by Cabaleiro et al. [81] who showed an enhancement up to 5% for 0.5 wt.% of sulfonic acid-functionalized graphene oxide nanoplatelets in ethylene glycol:water mixture at (10:90) wt.% at 323.15 K.

**Figure 9:**
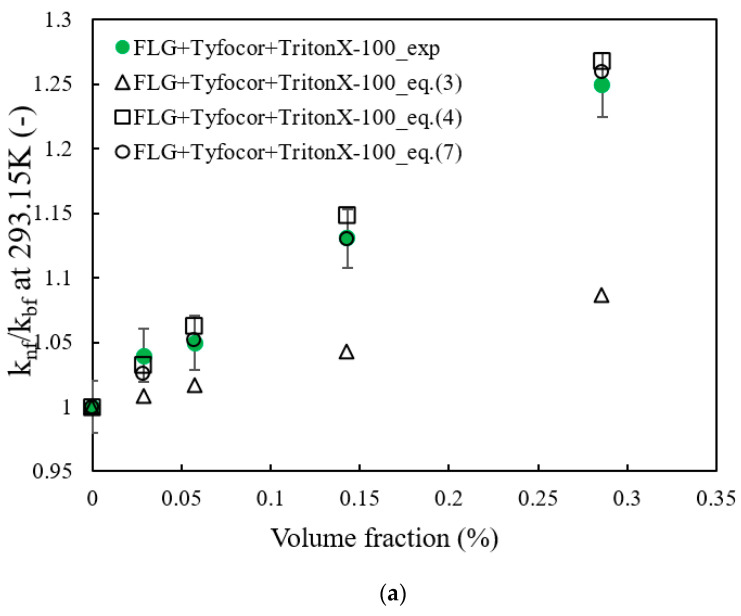

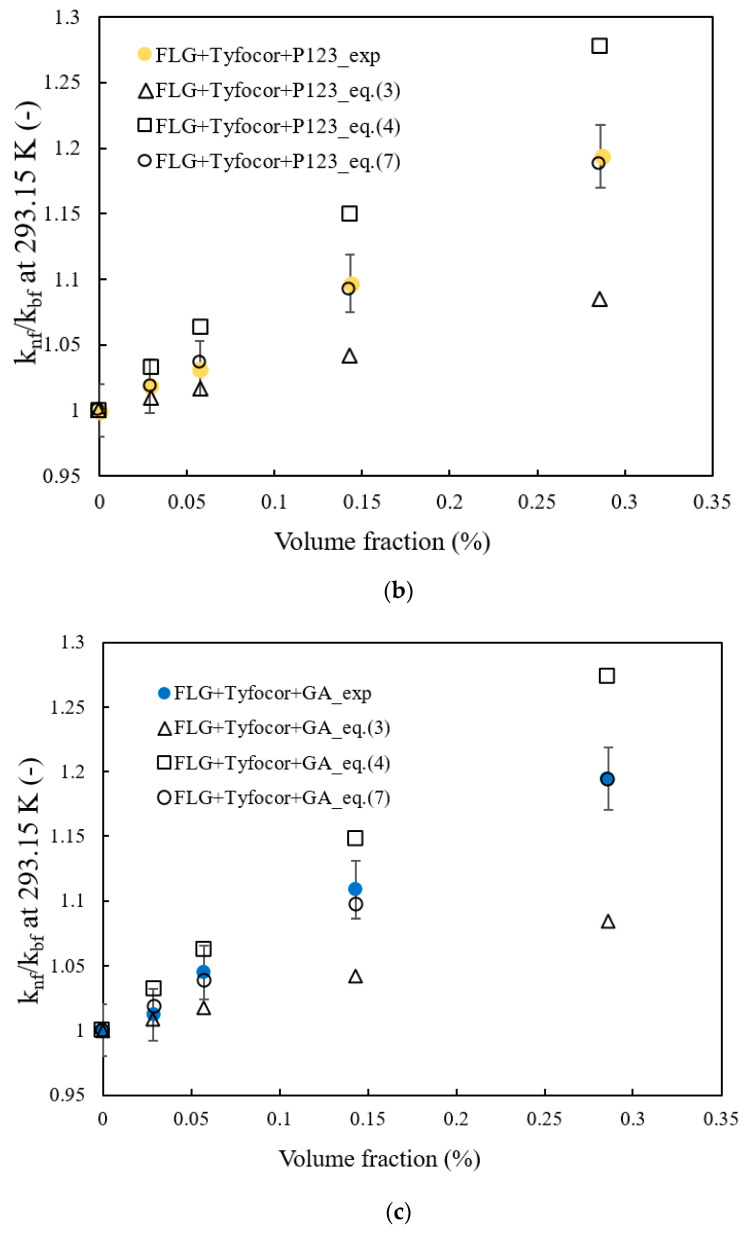
Thermal conductivity ratio of the FLG-based nanofluids over that of the corresponding base fluid at 293.15 K with TritonX-100 (**a**), Pluronic^®^ P-123 (P123) (**b**), and Gum Arabic (GA) (**c**)—Comparison between experimental data and theoretical models from Equations (3), (4) and (7).

Figure 9 highlights the thermal conductivity ratio of FLG nanofluids at 293.15 K as a function of the volume fraction of FLG. A linear increase in the thermal conductivity ratio with a slight difference in the slope for each used surfactant was observed. In this figure, as well as in the following models, the volume fraction of FLG, denoted φ and given by Equation (2), was obtained from the FLG mass concentrations $\varphi_{m}$, the density of each base fluid was evaluated from mixing rule using the density of each compound at 293.15 K and the density of FLG *ρ_np_* measured earlier, as in Equation (2).

(2) $$\varphi =\frac{\varphi_{m}\frac{\rho_{bf}}{\rho_{np}}}{\left(1-\varphi_{m}\left(1-\frac{\rho_{bf}}{\rho_{np}}\right)\right)}$$

Some models, presented in the following and that were previously used with graphene-based nanofluids or composites, are considered to analyze the observed thermal conductivity behaviors as the FLG content increases.

First, the upper Wiener bound or Parallel model [80] defined by Equation (3), was considered. In this equation, $k_{nf}$. and $k_{bf}$ correspond to the thermal conductivity of the nanofluid and base fluid, respectively, while $k_{np}$ is here the apparent thermal conductivity of the FLG, which is taken at 12 W m^−1^·K^−1^, as evaluated earlier from the guarded heat flow meter technique.

(3) $$k_{nf}=k_{np}\varphi +\left(1-\varphi \right)k_{bf}$$

Nan et al. [84] developed a thermal conductivity model for two-phase materials composed of dispersed particles in a liquid medium while taking the effect of interfacial resistance based on multiple scattering theory and the effective medium theory (EMT) model of Maxwell into account. Such a model also integrates the thermal conductivity along transverse and longitudinal axes of the particles. This model was previously used for the comparison purpose of the thermal conductivity of carbon-based nanofluids [85]. In the presence of graphene nanosheets, e.g., particles with large length and low thickness, it is admitted that the aspect ratio is quite high. By considering the fact that the thermal conductivity along transverse and longitudinal axes of the FLG nanosheets is much larger that of the thermal conductivity of the base fluid, the model of Nan et al. [84] takes the form of Equation (4), as proposed by [86]:

(4) $$k_{nf}=k_{bf}\left(\frac{1+\frac{2\varphi \left(\frac{k_{np}}{k_{bf}}\right)}{3}}{1-\frac{\varphi }{3}}\right)$$

In this equation, $k_{np}$ is the thermal conductivity of the FLG along the inplane direction, which depends on the graphene thickness and, consequently, the number of layers. This dependence of $k_{np}$ with graphene thickness t was modeled by molecular dynamic simulations in [87] and expressed by Equation (5) as followed:

(5) $$k_{np}=4058\times {\left(\frac{t}{3.4\times 10^{-10}}\right)}^{-1/2}$$

Based on the average thickness of 1.5 nm from TEM characterization, this leads to a thermal conductivity of the FLG along in plane direction of around 1930 W·m^−1^·K^−1^. Thus, by considering the significant thermal resistance effect at the interface between graphene and base fluid, the effective thermal conductivity of the FLG $k_{np}{}^{eff}$ can be written, as follows, Equation (6) [86].

(6) $$k_{np}{}^{eff}=\frac{k_{np}}{1+\frac{2R_{K}k_{np}}{L}}$$

where *L* is the average length of graphene taken here is 5 μm, as shown earlier from TEM, and $R_{K}$ the interfacial thermal resistance. It is assumed here that $R_{K}$ corresponds to the geometric average (60%/40%) of the interfacial resistance between the graphene nanosheets and water, it was fixed at 4.5 m^2^·K·W^−1^ considering the number of layers [88] and the interfacial resistance between the graphene nanosheets and propylene glycol is assumed to be close to that ethylene glycol 2.2 m^2^·K·W^−1^ [89]. This leads to a value of $R_{K}$ of 3.58 m^2^·K·W^−1^ for the used solvent (water and propylene glycol mixture). Consequently, from Equation (6), the obtained effective thermal conductivity is of around 67 W·m^−1^·K^−1^. This shows the influence of the interfacial thermal resistance, as this value is lower than the value given by Equation (5). This value is also far below the interfacial thermal resistance found from Equation (5) (1930 W·m^−1^·K^−1^) without considering the interfacial resistance between the FLG nanosheets and the base fluid, meaning that, for these nanosized platelets, the exposed surfaced within the fluid has great importance on the nanofluid behavior.

Finally, in addition to the thickness and interfacial thermal resistance dependence, the flatness ratio effect of graphene, denoted *η*, was also considered by Chu et al. [86] to take that contribution into account. Normally, thin nanosheets, like graphene, cannot be perfectly flat thermal platelets within the fluid in which they are dispersed, it is admitted that they rather adopt folded and corrugated shapes. Such a folded and wrinkled structure of FLG in solution reduces the effective length of the graphene nanosheets. This effect was shown to induce loss in the intrinsic thermal conductivity of graphene, which, consequently, may reduce thermal conductivity enhancements of graphene-based nanofluids. Chu et al. [86] proposed the following expression for thermal conductivity, which was used in [45] with graphene nanoplatelet nanofluids.

(7) $$k_{nf}=k_{bf}\left(\frac{3+\frac{2\eta^{2}\varphi }{\left[k_{bf}\left(\frac{2R_{K}}{L}+13.4\sqrt{t}\right)\right]}}{3-\eta \varphi }\right)$$

The comparison between the observed experimental thermal conductivity enhancement of the prepared FLG-based nanofluids at 293.15 K and theoretical models of Equations (3), (4) and (7) are presented in Figure 9. Equation (3) was not able to predict the evolution of thermal conductivity whatever the surfactant used. This result could be explained by the low thermal conductivity value used in this model. However, good agreement was observed for all nanofluid series with the model described by Equation (7). This correlation was obtained with only one adjustable parameter, namely the flatness ratio effect of graphene, *η* previously introduced, while the other values were used as defined before. The values of *η* are 0.75, 0.88 and 0.77 with TritonX-100, Pluronic^®^ P-123 and Gum Arabic, respectively. With these flatness ratios, the AAD (%) between data from the experiments and the models are 0.35, 0.11, and 0.21, respectively. The reported values of *η* are also in good agreement with the values previously reported with FLG [45,86,87,88,89]. This put into evidence the importance of the flatness ratio in addition to graphene nanosheet dimensions and thermal resistance at graphene interface in the thermal conductivity enhancement of graphene-based nanofluids. A relatively good agreement was also achieved with Equation (4), but with higher AAD of 4.11, 0.9, and 3.5%, with TritonX-100, Pluronic^®^ P-123, and Gum Arabic as surfactants, respectively.

## 4. Conclusions

Few-layer graphene (FLG) was produced following a mechanical exfoliation method that was assisted by tannic acid and then characterized by SEM, TEM, Raman spectroscopy demonstrating the excellent structural quality of the FLG nanosheets, and the efficiency of the exfoliation process. The density and the apparent thermal conductivity of these FLGs were also evaluated. The FLG nanosheets were used to develop nanofluids when considering a commercial heat transfer fluid based on a mixture of water and propylene glycol and different nonionic surfactants. The stability and the thermal conductivity of the produced nanofluids were experimentally characterized by multiple light scattering measurements and the transient short hot-wire method. The thermal conductivity studied was performed in the temperature range 283.15–323.15 K varying the mass content in FLG from 0.05 to 0.5%. The thermal conductivity of the prepared nanofluids was compared to several relevant models and it was shown the enhancement in thermal conductivity of graphene-based nanofluids is governed by combined effects, such as FLG size, thermal resistance at FLG interface, thickness, and their flatness ratio. Such reported thermal conductivity enhancement is promising in view of possible thermal applications.

## Nomenclature

ABS	Transmitted intensity
AAD	absolute average deviation [%]
DW	Deionized Water
*η*	flatness ratio effect of graphene
FFT	Fast Fourier TransformGum
GA	Gum Arabic
HFLG	highly crumpled few layer graphene
HRTEM	high-resolution transmission electron microscopy
FLG	few layer graphene
IFFT	Inverse FFT
L	Average length of graphene
PG	Propylene glycol
P123	Pluronic^®^ P123
*ρ*	Density
$R_{K}$	Interfacial thermal resistance
SEM	scanning electron microscopy
St. Dev.	standard deviation
TEM	transmission electron microscopy
T	temperature [K]
t	graphene thickness
t_0_	particles dispersion
TrX	Triton X-100
$\varphi_{m}$	mass fraction
φ	apparent mass fraction
φ	volume fraction
Subscripts/superscripts	
*eff*	effective
*nf*	nanofluid
*np*	nanoparticles
*bf*	base fluid
